# Epidemiology of Biliary Acute Pancreatitis—A Seven-Year Experience of a Large Tertiary Center

**DOI:** 10.3390/life15020139

**Published:** 2025-01-21

**Authors:** Andrei Vicențiu Edu, Mihai Radu Pahomeanu, Alexandru Olăreanu, Dana Gabriela Corbu, Andreea Ramona Treteanu, Alexandru Constantinescu, Vasile Șandru, Narcis Octavian Zărnescu, Lucian Negreanu

**Affiliations:** 1 Faculty of Medicine, Carol Davila University of Medicine and Pharmacy Bucharest, 050474 Bucharest, Romaniadanacorbu1@gmail.com (D.G.C.); andreea-ramona.treteanu0720@stud.umfcd.ro (A.R.T.);; 2 Internal Medicine & Gastroenterology Department, University Emergency Hospital of Bucharest, 050098 Bucharest, Romania; 3 Gastroenterology Department, Emergency Clinical Hospital of Bucharest, 014461 Bucharest, Romania; 4 Abdominal Surgery Department, University Emergency Hospital of Bucharest, 050098 Bucharest, Romania

**Keywords:** epidemiology, acute pancreatitis, biliary pancreatitis, gastroenterology, severity, length of stay, pancreatitis

## Abstract

(1) Introduction: One of the most common causes of acute pancreatitis is cholelithiasis, which is considered to be associated with female sex, older age, and recurrence. Our aim was to define a group of patients with B-AP to facilitate their diagnosis and management, while more judiciously using medical resources. (2) Materials and Methods: This retrospective, large cohort study, which was conducted by extracting data from the BUC-API registry, consisted of 1855 cases between 1 June 2015 and 1 April 2022. Each admission of the same patient was considered a separate case if it did not have signs of chronic pancreatitis. Severity and morphology were stratified according to the Revised Atlanta Classification. (3) Results: A total of 732 cases of B-AP were analyzed, with 92.5% occurring at the first attack. The median age was 65 years, with 61.9% of the patients being female. The majority (82.2%) were surgical cases, and the length of stay (LoS) was 7 days. There were 10.2% severe cases, with a mortality rate of 4%. (4) Discussion: We found positive associations between sex, age, recurrence, and morphology and biliary etiology. Compared with the general population, female sex and age over 65 years correlate better with a biliary etiology. In most scenarios, patients suffer from first attacks, with a lower probability of developing local complications. There was a tendency for biliary pancreatitis patients to be admitted to surgical wards.

## 1. Introduction

Acute pancreatitis represents one of the most common causes of gastrointestinal hospitalization. Being a disease characterized by the localized destruction and inflammation of the pancreas or a systemic inflammatory response. AP remains one of the most common gastroenterological conditions worldwide, with data from the Global Burden of Disease Study revealing a global incidence for pancreatitis of 2.75 million incident cases in 2021, representing an 8.5% increase compared to 2010 [1]. The incidence varies across countries and regions, ranging from a low incidence of 15.0 cases/100,000 in Denmark [2], 29.2 cases/100,000 in Romania to 83.7 cases/100,000 in Sweden [3]. Eastern Europe, in particular, shows high disability-adjusted life years, age-standardized incidence rates, and age-standardized mortality rates regarding AP [1].

The most common causes of AP are cholelithiasis, alcoholism, and hypertriglyceridemia. While alcoholism is more often encountered in Eastern Europe, the biliary etiology prevails in almost every other region [4] and seems to be twice as prevalent as the latter [5]. Furthermore, acute biliary pancreatitis is often more severe compared with alcoholic pancreatitis with a higher risk of complications [6]. While mild AP typically resolves on its own and has a positive prognosis, severe cases have high mortality rates ranging from 20% to 40% [7]. Aside from its intrinsic mortality, AP is a significant risk factor for pancreatic neoplasia, a condition that is diagnosed late in evolution and has a very low 5-year survival rate [8,9].

There is a high degree of heterogeneity in the management of B-AP, with multiple guidelines and no clear consensus in this area [10]. Several recent meta-analyses have shown that early cholecystectomy might lower the rates of complications and recurrence and could shorten the length of stay (LoS) [11,12]. Although there is a clear association between biliary pancreatitis and female sex, older age, and recurrence [13,14], there seems to be no difference among etiological groups regarding the outcome of AP [15].

The aim of this study was to define the group of patients with B-AP by clinical and demographic criteria, especially to determine whether there are any associations between age and biliary etiology. The following associations were investigated: B-AP with sex, severity, cost, recurrence, ward of care, LoS, intensive care unit (ICU) admission, and LoS-ICU.

## 2. Materials and Methods

This was a retrospective cohort study that used data from the BUC-API registry. This registry is a single-center repository that includes 2042 consecutive cases of various pancreatic pathologies (acute pancreatitis, recurrent acute pancreatitis, and acute-on-chronic pancreatitis).

The registry was developed with the approval of the ethics committee of the University Emergency Hospital of Bucharest (protocol code 68629/19, date of approval: 18 October 2024). All patients provided informed consent for the use of their data for medical research prior to admission to the hospital. The study followed the ethical guidelines stated in the 1975 Declaration of Helsinki and those from the STROBE guidelines.

For this particular study, any admission for the same patient was considered a separate case. The electronic health records of the University Emergency Hospital of Bucharest were used to identify cases. ICD-10 specific codes for pancreas-related diseases (K85, B26.3 and B25.2) were used to refine the search among all discharges from the hospital that occurred between 1 June 2015 and 1 April 2022.

**Figure 1:**
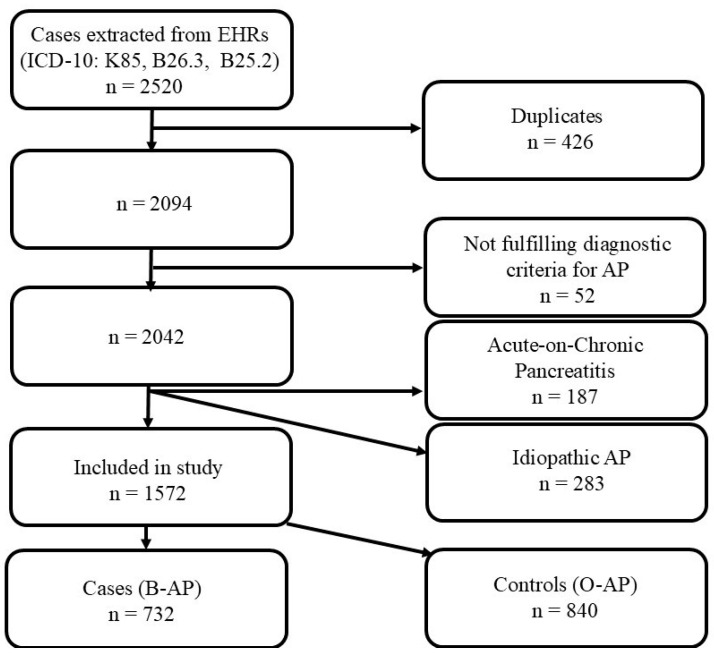
Cases flowchart.

Initially, there were 2520 extracted cases from EHRs, those cases were assessed by trained medical staff, and then, 426 were excluded, because they were duplicates, together with other 52 cases that were miscoded, because they did not fulfill 2 out of 3 diagnostic criteria for AP [16]. From the remaining 2042 cases, after thorough inspection, another 187 cases that were in fact acute-on-chronic pancreatitis were excluded from the study, as they fulfilled both evidence-based medicine imaging diagnostic criteria of chronic pancreatitis [17] (atrophy, fibrosis, dilated ducts, calcifications, and fibrosis) and those of AP [16]. The final number of cases included in the analysis was 1572, after excluding idiopathic AP cases in order to simplify the statistical analysis and the conclusions of this study. (See cases flowchart in Figure 1).

In order to establish the etiology, the criteria stated by Besselink et al. [16] were used. Any patient who had at least one of the following criteria for biliary–etiological AP was included: ALT > 150 U/L within the first 48 h from admission and imaging (ultrasound or CT) evidence of biliary pathology that might influence pancreatic secretion and/or known gallstone disease without another evident etiology was considered as part of the B-AP group. To simplify the statistical analysis, all other known etiology cases were merged into a single group that was compared with the biliary cases.

Any case of AP without signs of chronic pancreatitis that was previously admitted to the hospital within the time frame of the BUC-API registry and/or was known with previous episodes of AP was considered a recurrent case. Severity and morphology (local complications) were stratified according to the Revised Atlanta Classification [18].

The decision to be admitted to a particular ward was made by a multidisciplinary council in the ER comprising representatives of emergency medicine, a gastroenterologist, a surgeon, and a radiologist.

For this study, the data were organized using Microsoft 365 © (Microsoft Inc., Redmond, WA, USA) and Google Docs © (Alphabet Inc., Mountain View, CA, USA). To observe the general characteristics of the cohort, frequency tests were employed. To examine the correlation between two categorical variables, we employed the Pearson chi-square test and Phi Cramer’s V test, receiver operating curve and its derivatives, and multinomial logistic regression. The Mann–Whitney U-test was used to examine the correlation among continuous and categorical variables. SPSS Statistics version 29.0.0.0 © (IBM Inc., Armonk, NY, USA) was used for all the statistical analyses and charts from this study. Zotero 6 © (Corporation for Digital Scholarship, Vienna, VA, USA) for Windows and Zotero Connector for Google Chrome were used to manage the references and bibliography.

## 3. Results

### 3.1. Population Characteristics

In the GP population of the AP cases, the first attack prevails (82.8%), which is the same as that in the B-AP cases (92.5%). The median age was 57.0 years in the GP group but was greater in the B-AP group (65.0 years). In the GP group, there was a minority of females; whereas, in the B-AP subgroup, they represented the majority (40.8% vs. 61.9%). With respect to the ward of care, in the GP, there were a minority of surgical cases; whereas, in the B-AP cohort, they were the most common (49.3% vs. 82.2%).

The morphology of the GP group was predominantly interstitial edema (38.5%), similar to that of the B-AP (37.4%), but there were fewer data available in the B-AP group (30.3% vs. 32.4%) and more cases of normal pancreas at discharge than in the GP group (14.8% vs. 20.2%). Both the GP and B-AP healed outcomes at discharge were highly prevalent (83.0% vs. 83.7%), and there were similar mortality rates between the two aforementioned pathologies (5.8% and 4.0%, respectively).

An identical median LoS between the GP and B-AP patients (7.0 days) was observed. Most of the GP cases had biliary (39.5%), alcoholic (33.9%), or idiopathic (15.2%) etiologies. There were 12.9% severe cases in the GP group and a mildly lower rate of 10.2% in the B-AP group. A slightly lower ICU admittance rate in the B-AP group than in the GP group (8,7% vs. 9.6%) was found. The rates of rurality were similar for both GPs (27.3%) and B-APs (27.6%) (Table 1 and Table 2).

### 3.2. Age

**Figure 2:**
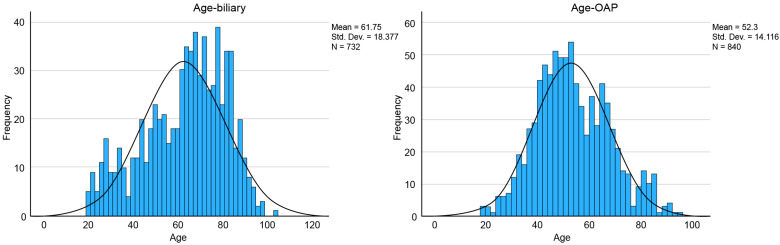
Age distribution amongst the two groups.

Mann–Whitney U-tests were used to determine whether there were any differences in etiology or age. The results indicated that B-AP patients were significantly older than O-AP patients were, U = 202,354.5, Z = −11.7, *p* < 0.01. The median age was 65.0 years (IQR = 17.0) in the B-AP and 51.0 years (IQR = 20.7) in the OAP (Table 2, Figure 2).

**Figure 3:**
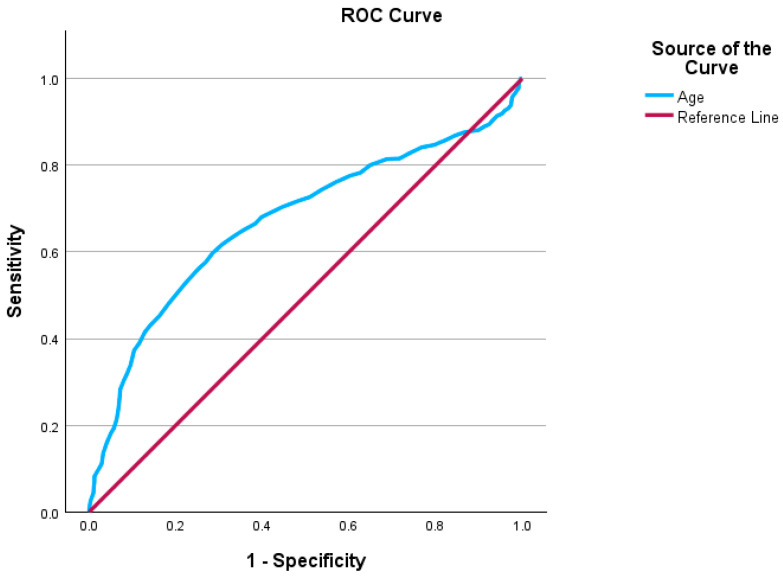
ROC for age.

A receiver operating characteristic (ROC) curve analysis yielded an AUC of 0.67 (95% CI [0.64, 0.70]), suggesting moderate statistical discriminatory ability in regard to age as a predictor of B-AP. The optimal cut-off for the model was determined using Youden’s index (J). As such a J = 0.31, with a sensitivity of 59.7% and a specificity of 71.5% at a threshold of 60.50 years (Figure 3).

### 3.3. Gender

To compare the distributions of sex in the two etiology groups, a chi-square test of independence was run. The aforementioned test revealed a significant difference between the two variables (X2(1) = 292.6, *p* < 0.01). A Cramer V of 0.43 implies that there is a medium–strong magnification association. As a post hoc test, we obtained an adjusted standardized residual (ASR) of +17.1 with respect to women with B-AP, which means that there is an important difference from the expected frequencies, with women being more prone to developing B-AP (Table 2).

### 3.4. Recurrence

The chi-square test revealed that the number of previous attacks of AP without chronicization differed significantly (X2(1) = 102.1, *p* < 0.01). Cramer’s V of 0.26 suggests a medium-strength association. When ASR was used as a post hoc test, the first attack of B-AP differed in frequency from the expected ASR of +10.1 (Table 2).

### 3.5. Outcome

To check for any distinction between outcomes at discharge and etiology, a chi-square test of independence was used. There were statistically significant differences (X2(4) = 45.6, *p* < 0.01). Cramer’s V (V = 0.17) suggests a medium-strength association. In post hoc testing, differences in the expected frequency with respect to transfer (ASR = +5.5 in the B-AP group) and discharge-at-will (ASR = −4.1 in the B-AP group) were obtained, implying that B-APs are more likely to be transferred to another unit and less likely to be discharged at will (Table 2).

### 3.6. Morphology

A chi-square test of independence revealed a statistically significant difference (X2(4) = 55.3, *p* < 0.01) between morphology and etiology. Cramer’s V (V = 0.18) indicates a medium-strength association. Determining the ASR via post hoc tests revealed differences in the expected frequency of the B-AP group: normal pancreas (ASR = +4.9), local fluid complications (ASR = −5.5), and no data (ASR = +2.4) (Table 2).

### 3.7. Ward of Care

There was a noticeable difference between the ward of care and etiology using a chi-square test of independence (X2(1) = 569.6, *p* < 0.01). Cramer’s V (V = 0.60) also revealed a strong association. By calculating the post hoc ASR, we observed a meaningful difference in the expected frequency with respect to B-APs and surgical wards (ASR = +23.9) (Table 2).

### 3.8. Multinomial Logistic Regression for the Model

A multinomial logistic regression analysis was performed to observe the relationship between etiology and the following predictor variables: age, sex, recurrence, and ward of care. The model was statistically significant, indicating that it was able to differentiate among the etiologies based on the predictor variables X^2^ (4) = 708.9, *p* < 0.01. For B-AP compared with O-AP, all the variables were significant predictors. Each year increase in age was associated with a 3% increase in the likelihood of suffering from B-AP (Exp(B) = 1.03, *p* < 0.01, 95% CI [1.03, 1.04]). Sex was a significant predictor, as males were 82% less likely to suffer from B-AP (Exp(B) = 0.18, *p* < 0.01, 95% CI [0.14, 0.24]). The recurrence was an important predictor, as those suffering from their first attack of AP were 110% more likely to suffer from B-AP (Exp(B) = 2.10, *p* < 0.01, 95% CI [1.38, 3.21]). The ward of care was also a predictor, as those that were cared for in surgical wards were 14 times more likely to suffer from B-AP (Exp(B) = 15.14, *p* < 0.01, 95% CI [11.35, 20.19]).

### 3.9. Other Aims

No statistically meaningful differences between etiology and the following outcomes were observed: severity (X2(2) = 2.7, *p* = 0.26), ICU admission rate (X2(1) = 0.4, *p* = 0.84) or LoS-ICU (U = 2136.0, Z = −0.6, *p* = 0.54), rurality (X2(3) = 2.8, *p* = 0.43), or LoS (U = 298,934.0, Z = −0.9, *p* = 0.34) (Table 2).

## 4. Discussion

The findings suggest a correlation between sex and etiology, as it appears that female sex is correlated with B-AP with respect to O-AP. This finding is in accordance with another study [19] that reported a greater female predilection for B-AP than for alcoholic AP. We do not find this surprising, as it is well established [20] that estrogen heightens the risk for developing cholesterol gallstones by augmenting the cholesterol saturation of bile secretion.

B-AP was more common in patients over 60 years of age. This contradicts the data from Cho et al. [21], as they were not able to find any significant differences regarding age. However, in other cohorts [13,19], a similar association between older age and B-AP was found. This heterogeneity can be explained by the different numbers of patients included (153 in Cho et al.) and greater than 700 patients in the other groups.

B-APs are associated with a greater probability of a normal pancreas during imaging investigations; whereas, O-APs are more likely to develop with fluid collections (APFCs and pseudocysts). This finding correlates with the findings of Du et al. [22], who reported a lower probability of developing local complications in B-AP patients, and with the findings of another paper [21], who reported significant differences with regard to pseudocyst development, which is more often found in alcoholic AP than in B-AP. While there are few reports in the literature regarding local complications stratified by etiology, our results may facilitate a timely and definitive solution involving cholecystectomy and/or ERCP in B-AP patients, as ERCP remains a method of choice in common bile duct lithiasis [23].

The study data showed that B-AP is more commonly seen as a first attack of AP. In the literature [24,25], there seems to be a large consensus that B-AP is associated with a single episode; whereas, other etiologies (mainly alcoholic) may be linked with recurrence. This effect is most likely caused by the instrumentation of the biliary tract in the B-AP with a definitive solution, as stressed by other peer researchers [26,27].

With respect to the ward of care, any previous analysis regarding the correlation between the type of ward and etiology were hard to find, as we observed a tendency for biliary cases to be admitted to surgical wards instead of gastroenterological wards. Nevertheless. Several studies that address this problem are suggesting that admission directly to surgical wards could lower the recurrence rate, shorten LoS, and lower costs [28]. Another retrospective study [29] revealed that patients with B-AP who were cared for by surgeons had a lower count of consulting services, laboratory tests, and antibiotics. As such, it is considered that admittance to surgical wards might translate to better care for B-AP patients and a more rapid, definitive solution, which will probably also translate to a lower cost of care.

In a previous study [30] on the same population but focusing on alcohol-induced AP, the other most prevalent etiology, it was found that the epidemiology of those patients differs a lot from those with B-AP. The first ones are more probable to be middle-aged male patients with recurrent AP. As such, there could be a better stratification of the etiology based also on demographic and historical factors in addition to medical ones.

Exploring the outcome, we find that there is a significantly greater probability for B-AP to be transferred; whereas, for O-AP, there is a greater probability of discharge-at-will. As we could not find any data on this topic stratified by transfer and/or discharge, it should be considered that both of these outcomes might be influenced by the fact that this study is unicentric. More multicenter studies on this topic are required, as this might be a local effect and not a disease-related effect. No statistically relevant differences with respect to mortality could be found.

Although not part of the aim of the study, we cannot end this paper without reminding that a large part of B-AP patients can suffer a second attack of AP after the instrumentalization of the common bile duct with ERCP for choledochal lithiasis extraction. The post-ERCP pancreatitis in this particular indication was very well described by previous studies in the literature [31].

In this study, it was a lack of any significant differences regarding severity, rurality, LoS, ICU admission, or ICU-LoS. It was difficult to determine whether this lack of difference might be caused by limitations in the study design, such as its retrospective design, the unicentricity of the study, missing data from EHRs, and/or any potential confounding variables that could not be accounted for. Although rare, it is possible that this study may have missed some AP cases related to biliary heterotopia of the pancreatic tissue [32], as we have not accounted for them. Other limitations of this study can arise from the fact that we have not checked for any surgical or endoscopical interventions required in order to solve the biliary pathology.

As strong points of this study, the large number of cases analyzed and the lack of selection bias stand out, as all cases were consecutive admissions with clear inclusion and exclusion criteria and long durations.

## 5. Conclusions

The ROC and multinomial logistic regression showed us a possible reliable future model for predicting biliary etiology based solely on demographic factors. This should include in the analysis: age, sex, recurrence, and ward of care. Based on our study, the typical sketch of a biliary acute pancreatitis patient should be female in her sixties at her first attack of AP admitted to a surgical ward. Other factors that might contribute to the predictive model are pancreatic morphology and outcome at discharge.

## Figures and Tables

**Table 1:** Population characteristics.

AP Cases	*n* = 1855
Recurrence	
First known attack	1536 (82.8%)
Recurrent AP	319 (17.2%)
Age (years)	
Median	57 (IQR = 26.0)
Mean	56.9 (SD = 17.1)
Length of stay (LoS)	
Median	7.0 (IQR = 6.0)
Mean	8.8 (SD = 7.8)
Daily cost of hospitalization (EUR)	
Median	185.3 (IQR = 87.0)
Mean	433.2 (SD = 3446.7)
Etiology	
Biliary	732 (39.5%)
All other known causes	840 (45.3%)
Idiopathic	283 (15.2%)
Gender	
Male	1098 (59.2%)
Female	757 (40.8%)
Severity	
Mild	954 (51.4%)
Moderately severe	677 (36.5%)
Severe	224 (12.1%)
Morphology	
Interstitial	715 (38.5%)
Normal pancreas	274 (14.8%)
Fluid collections	179 (11.4%)
Necrosis	65 (4.1%)
No data	562 (30.3%)
Outcome	
Healed/ameliorated	1540 (83.0%)
Discharged at will	116 (6.3%)
Deceased	108 (5.8%)
Transferred	79 (4.3%)
Stationary	12 (0.6%)
ICU	
No	1676 (90.4%)
Yes	179 (9.6%)
Ward of care	
Gastroenterology	941 (50.7%)
Surgery	914 (49.3%)
Rurality	
Urban	1332 (71.8%)
Rural	507 (27.3%)
No data	16 (0.9%)

**Table 2:** Comparison of elements between the two etiological groups. All statistically significant *p*-values are marked with *.

	**B-AP (*n* = 732)**	**O-AP (*n* = 840)**	***p*-Value**
Severity			
Mild	394 (53.8%)	421 (50.1%)	0.25
Moderately severe	263 (35.9%)	316 (37.6%)	
Severe	75 (10.2%)	103 (12.3%)	
ICU			
No	668 (91.3%)	769 (91.5%)	0.84
Yes	64 (8.7%)	71 (8.5%)	
Ward of care			
Gastroenterology	130 (17.8%)	656 (78.1%)	<0.01 *
Surgery	602 (82.2%)	184 (21.9%)	
Gender			
Male	279 (38.1%)	675 (80.4%)	<0.01 *
Female	453 (61.9%)	165 (19.6%)	
Outcome			
Healed/ameliorated	613 (83.7%)	705 (83.9%)	<0.01 *
Stationary	6 (0.8%)	5 (0.6%)	
Transfer	57 (7.8%)	16 (1.9%)	
Discharge at will	27 (3.7%)	73 (8.7%)	
Deceased	29 (4.0%)	41 (4.9%)	
Recurrence			
First attack	677 (92.5%)	612 (72.9%)	<0.01 *
Recurrence	55 (7.5%)	228 (27.1%)	
Morphology			
Interstitial	274 (37.4%)	349 (41.5%)	<0.01 *
Fluid collections	49 (6.7%)	130 (15.5%)	
Necrosis	24 (3.3%)	41 (4.9%)	
Normal pancreas	148 (20.2%)	94 (11.2%)	
No data	237 (32.4%)	226 (26.9%)	
Rurality			
Urban	526 (71.9%)	588 (70.0%)	0.43
Rural	202 (27.6%)	241 (28.7%)	
No data	4 (0.5%)	11 (1.3%)	
Age (years)			
Mean	61.8 (SD = 18.4)	52.3 (SD = 14.1)	<0.01 *
Median	65.0 (IQR = 27.0)	51.0 (IQR = 20.7)	
Length of stay (days)			
Mean	8.4 (SD = 5.7)	9.0 (SD = 8.4)	0.34
Median	7.0 (IQR = 6.0)	7.0 (IQR = 5.0)	
ICU Length of stay (days)			
Mean	5.0 (SD = 4.7)	5.4 (SD = 5.9)	0.54
Median	2.5 (IQR = 5.0)	4.0 (IQR = 4.0)	

## Data Availability

Data available upon reasonable request from the corresponding author.
